# The diagnosis and management of neuropathic pain in daily practice in Belgium: an observational study

**DOI:** 10.1186/1471-2458-7-170

**Published:** 2007-07-24

**Authors:** Guy Hans, Etienne Masquelier, Patricia De Cock

**Affiliations:** 1 Multidisciplinary Pain Center, Antwerp University Hospital (UZA), Edegem, Belgium; 2 Centre de Douleur, Cliniques Universitaires UCL de Mont Godinne, Yvoir, Belgium; 3 Service de médecine physique et de réadaptation motrice, Cliniques Universitaires UCL Saint.-Luc, Brussels, Belgium; 4 Medical Department Pfizer Belgium, Brussels, Belgium

## Abstract

**Background:**

This open, multicentre, observational survey investigated how physicians diagnose neuropathic pain (NeP) by applying the Leeds Assessment of Neuropathic Symptoms and Signs (LANSS) scale, and how neuropathic pain conditions are managed in daily practice in Belgium.

**Methods:**

Physicians were asked to complete the Leeds Assessment of Neuropathic Symptoms and Signs (LANSS) scale for diagnosing NeP, and to fill out a questionnaire regarding the management of NeP, together with a questionnaire evaluating the impact of pain on sleep and daily life. Data on 2,480 pain patients were obtained. A LANSS score ≥ 12 (meaning NeP is most probably present) was reported for 1,163 patients. Pathologies typically associated with NeP scored above 12 on the LANSS scale, contrary to pathologies generally considered as being of non-neuropathic origin.

**Results:**

Over 90% of the patients with a LANSS score ≥ 12 reported that the pain impaired sleep. A high impact on social, family and professional life was also recorded. Additional examinations were performed in 89% of these patients. Most patients were taking multiple drugs, mainly paracetamol and non-steroidal anti-inflammatory drugs, indicating that physicians generally tend to follow treatment guidelines of chronic nociceptive pain, rather than the specific ones for NeP. Specific neuropathic guidelines rather recommend the use of anti-epileptic drugs, tricyclic antidepressants or weak opioids as first-line treatment.

**Conclusion:**

In our survey, application of the LANSS scale lead to pronounced treatment simplification with fewer drug combinations. Awareness about NeP as well as its specific treatment recommendations should be raised among healthcare providers. We concluded that the LANSS screening scale is an interesting tool to assist physicians in detecting NeP patients in routine clinical care.

## Background

Neuropathic pain (NeP) has been defined by the International Association for the Study of Pain (IASP) as pain that is initiated or caused by a primary lesion or dysfunction in the nervous system [1]. NeP often manifests as spontaneous pain (e.g. burning, throbbing, shooting, electric shock sensations), as well as pain that is provoked by stimuli that are normally not painful (allodynia), or that elicit an exaggerated response to a painful stimulus (hyperalgesia) [2]. NeP is thought to be present in ~25% of chronic pain patients [3], but remains frequently undiagnosed. Hence, treatment may often be inadequate [4,5]. NeP is often associated with comorbid conditions such as poor sleep, depression, mood disturbances, and a lowered quality of life (QoL) [6].

Common causes of NeP include diabetes mellitus, cancer, herpes zoster, trigeminal neuralgia, complex regional pain syndrome (CRPS), alcohol abuse, multiple sclerosis (MS) and other [7]. NeP is notoriously difficult to treat and tends to be refractory to the analgesics commonly employed for treating nociceptive pain (NocP), such as paracetamol and non-steroidal anti-inflammatory drugs (NSAIDs) [4,8]. In contrast, antiepileptic (anticonvulsant) drugs (AEDs), antidepressants, and some opioids have proven efficacy against several manifestations of NeP [4,9,10].

However, a major hurdle in establishing a correct diagnosis and hence to provide the patient with the appropriate therapy is the lack of unambiguous diagnostic criteria for distinguishing NeP from NocP. Physicians often rely on the clinical diagnosis that may be complemented with exams such as radiological examinations (RX, CT scan or MRI), electromyography (EMG), or a somatosensory evoked potential test. Recently, the Leeds Assessment of Neuropathic Signs and Symptoms (LANSS) pain scale was developed and validated to provide clinicians with a practical instrument for discriminating NeP from NocP in daily practice [11]. The LANSS pain scale consists of two parts: (1) a 5 item pain questionnaire that has to be completed by the patient, with queries about the nature of the pain, and (2) a simple sensory testing part for assessing the presence of allodynia and hyperalgesia that has to be carried out by the physician [11]. The LANSS scale has been previously employed in various pain states, such as post-thoracotomy pain [12], low back pain [13], fibromyalgia [14,15], and head and neck cancer [16].

The objective of this survey was to increase awareness of NeP among Belgian physicians and to determine whether a screening scale would be helpful in making the correct diagnosis. Furthermore, we aimed to assess how NeP is managed in Belgium, and how this condition affects the quality of sleep and daily life in the patients. To our knowledge, this is the first study to report on the use of a pain scale in the diagnosis of NeP on a large scale in real-life clinical practice.

## Methods

### Study design

One hundred and seventy seven general practitioners (GP) and 97 specialists (mostly rheumatologists, pain specialists and neurologists) participated in this study. In Belgium (pain) patients can directly consult any specialist of their choice, without prior referral by their GP. Because GP's in Belgium often do not act as frontline physicians for pain conditions – with pain patients immediately contacting the specialists they consider appropriate for solving their problem – we believe that there is no major difference in patient population nor pain characteristics in the specialists' reception from those in GPs'. The study was approved by the local ethics committee of the Antwerp University Hospital (UZA). Written informed consent was obtained from *all *patients before their inclusion in the study.

Participating physicians were instructed to document 15 *consecutive *patients presenting with symptoms of sub-acute (between 3 and 6 months of duration) and chronic (> 6 months) pain in their practice, irrespective of the type of pain (nociceptive or neuropathic). Pregnant and breastfeeding women, and women who were planning to become pregnant in the near future, were excluded from the study. Age, gender, and duration of pain of all patients were recorded. The possible underlying cause of the pain was also documented by the physician.

The physicians were asked to fill out the LANSS questionnaire (characterization of the pain true 5 questions), as well as to perform the two included items for sensory testing: (1) allodynia and (2) altered pin-prick threshold. Allodynia was judged to be present when pain was elicited by gently stroking a piece of cotton wool over the painful area and when normal sensation was experienced in the control site. Hyperalgesia was judged to be present when pin-prick testing elicited an exaggerated painful response at the painful site compared with the control site. Validation of a Dutch and French version of the original (English) LANSS-scale was performed before the start of the study, in accordance to how this validation was performed for other languages [17,18]. Translation and back-translation method was used to adapt the LANSS into Dutch and French. In this process, the scale was first translated and culturally adapted into Dutch and French by a translator who spoke English fluently (conceptual equivalence approach). The scale was then back-translated into English by a native English speaker who had not seen the original English version. The back-translated English version was then compared by several experts with the original LANSS in English. Bilingual fluency was required for all translators involved in this process.

For the patients who had a LANSS scale pain score ≥ 12 (meaning NeP is most probably present) the physicians filled out an additional questionnaire providing more detailed information on (1) previously performed and future planned treatment options for the pain condition, (2) any additional tests that were performed for confirming the diagnosis and (3) the impact of the pain symptoms on sleep and activities of daily living. Although useful information regarding treatment and functional status could have been generated if *all *patients would have completed this additional questionnaire, due to practical reasons (work load of participating physicians) it was opted by the investigators not to obtain this additional information in patients with LANSS-scores < 12. Sleep was assessed in a quantitative way by using a visual analogue scale for sleep quality (VAS) offering a score between 0 (no sleep disturbance) to 10 (maximal sleep disturbance) for sleep disturbance during the past 24 h. In addition, sleep was assessed qualitatively by asking the patients which specific aspects of sleep were disturbed (inability to fall asleep, frequent interruptions of sleep, non-restorative sleep and premature awakenings). Finally, patients were asked if their pain had an impact on following aspects of daily life: family life, social activities, professional life and leisure.

### Statistical analysis

Data analyses were based on descriptive statistics, including percentages, medians, means, ranges and standard deviations. Percentages were calculated based on all values for that particular question (missing excluded). Analyses were performed on the total patient group as well as the LANSS pain score subgroups (cut-off value at 12). Comparison of parameters (duration of pain, age) between LANSS pain score subgroups was carried out using t-test statistics. Fisher's exact test was employed for assessing differences in gender and type of pain. All statistical tests were interpreted at the 5% significance level (two-tailed). In addition, odds ratios, together with 95% confidence intervals, were calculated for the patients with only one underlying cause of pain to investigate the influence of the underlying pathology on the LANSS pain scores. Patients reporting multiple underlying causes of pain were excluded from the odds ratio analysis to avoid confounding effects of the various pathologies on the LANSS score.

## Results

### Total patient population

In total 2,480 pain patients were enrolled in the study by 177 general practitioners (GPs) and 97 specialists. The total number of patients that could have been expected was not reached since 63 physicians (28 GP's and 35 specialists) failed to fulfil the inclusion target, and a number of questionnaires (n = 493) had to be discarded due to incompleteness of the data. Reasons for these failures are probably the work load and the strict time schedule of this protocol. Around two thirds of the patients (n = 1,649) were treated by a GP, while 831 patients (34%) were being treated by a specialist. More than half of the participating pain patients (59.6%) were women. The mean age of the patients was 58.2 years (standard deviation, SD, = 15.7). The average duration of the pain amounted to 3.0 years (SD = 4.4), with a range from less than three months to 60 years (diabetic neuropathy). Almost one third of the patients (28%) reported their pain having lasted from six months to three years.

### Underlying pathologies

**Figure 1:**
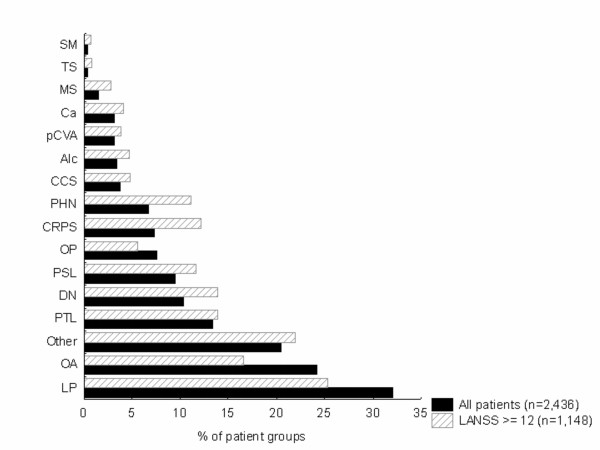
**Identified causes of pain**. Possible underlying causes of pain in (1) all patients pooled (n = 2,436) and (2) the patients with a LANSS pain score ≥ 12 (n = 1,163). Patients may have multiple underlying pathologies. *DN: diabetic neuropathy, TS: trigeminal syndrome (neuralgia); Ca: cancer, LP: lumbar pain, OP: osteoporosis; MS: multiple sclerosis, PHN: post-herpetic neuralgia, CRPS: complex regional pain syndrome; SM: Syringomyelia; CCS: carpal canal syndrome; pCVA: post-cerebrovascular accident; Alc: alcohol abuse; PSL: post-surgical lesions; PTL: post-traumatic lesion; OA: osteoarthritis*.

Data on the underlying pathology were obtained for 2,436 out of 2,480 pain patients (98%). A total of 65.2% of the patients had one underlying cause of pain, while in 25.4% of patients two underlying disorders could be identified. Three or more pathologies were reported by 9.4% of the patients. The most prevalent causes of pain consisted of lumbar pain (32.1% of patients), followed by osteoarthritis (OA) (24.2%), post-traumatic lesions (13.4%), diabetes mellitus (10.4%), post-surgical lesions (9.5%), osteoporosis (7.6%), complex regional pain syndrome (CRPS) (7.3%), and post-herpetic neuralgia (6.7%) (Fig. 1). The most frequently described combination of diagnoses was lumbar pain in combination with OA (reported in 6.0% of patients), followed by lumbar pain combined with post-surgical lesions (1.9% of patients). It Is important to mention that there were no statistical differences between the pathologies diagnosed by the GP's or the specialists, with both physicians groups reporting similar percentages for the different pathologies.

### Type of pain symptoms

Spontaneous pain was present in 96.5% of the patients. A total of 61.5% of patients seeking medical help reported a combination of spontaneous and provoked pain symptoms (61.5%), while 37.8% reported spontaneous pain as their only symptom. Finally, 0.7% of patients reported only provoked pain symptoms. Spontaneous pain was mainly described in terms of throbbing pain (61.3%), a burning sensation (47.9%), or abnormal and disagreeable (painful) sensations (dysaesthesia) (47.8%). Less frequent manifestations of spontaneous pain comprised cutting-lacerating pain (31.8% of patients), electric shock sensation (22.8%), stab sensation (18.8%), and abnormal but not disagreeable sensation (paraesthesia) (17.3%). The majority of the patients with spontaneous pain symptoms (57.7%) identified two or three terms to describe their symptoms. The combination of a burning sensation, throbbing pain and dysaesthesia was the most frequently reported combination (6.4% of the patients with spontaneous pain), followed by a burning sensation together with dysaesthesia (4.5%), throbbing pain with dysaesthesia (4.3%), and throbbing pain in combination with cutting-lacerating pain symptoms (4.0%).

Evoked pain was mostly present as allodynia (40.3%), hyperalgesic symptomatology only (10.3%), or mixed allodynic/hyperalgesic symptoms (47.0%). Touch-evoked allodynia was most common (71.7% of all patients suffering from provoked pain). These allodynic symptoms were induced by several triggers, such as contact with clothes (38.8%), or allodynia in the shower or in bath (15.2%), followed by painful symptoms resulting from wind blowing against the face (12.5%) and during shaving (5.4%). Pin-prick hyperalgesia was found to be the most common form of hyperalgesia, being present in more than half (52.5%) of all patients suffering from hyperalgesic symptoms. Other forms of hyperalgesia were reported in 2.5% of the patients. Once more, as observed previously with spontaneous pain, a majority of patients (61.6%) reported more than one subtype of provoked pain. The most frequently reported combinations of provoked pain symptoms were touch-evoked allodynia combined with pin-prick hyperalgesia (12.5% of the patients with provoked pain symptoms), followed in second place by touch-evoked allodynia combined with allodynia induced by clothing and pin-prick induced hyperalgesia (11.7%).

### Subgroups of patients: LANSS scale pain score <12 versus LANSS scale pain score ≥ 12

Of the total number of patients enrolled in this survey for whom a LANSS pain score was recorded (n = 2,464), 1,163 patients (47.2%) presented with a LANSS score of 12 or more (LANSS ≥ 12 group), which strongly suggests the presence of a pain component of neuropathic origin [11]. Demographic data were similar across the subgroups, showing no significant differences in age (mean 57.7 yrs, SD = 15.5 vs. 58.6 yrs, SD = 15.8; NS), gender (58.5% vs. 60.5% women; NS), or duration of pain (mean 3.1 yrs, SD = 4.1 vs. 2.9 yrs, SD = 4.7; NS) between the LANSS ≥ 12 group and the patients with a LANSS score < 12 (LANSS < 12 group) respectively. We did not find any significant association between LANSS score and duration of the pain in either subgroup (data not shown).

### LANSS scale pain scores in relation to the underlying pathologies

**Figure 2:**
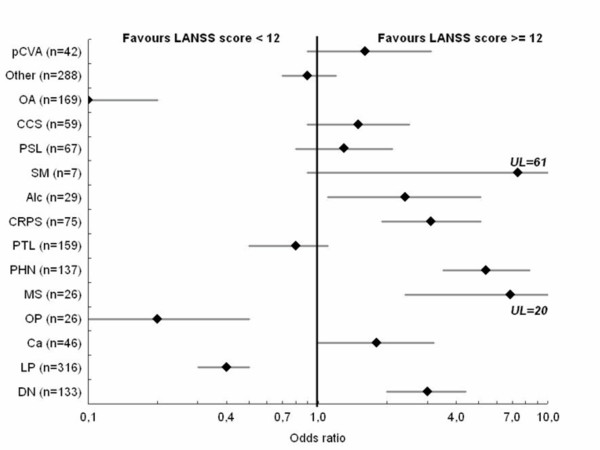
**Calculated Odds ratios**. Odds ratios for the various pathologies having a LANSS score ≥ 12 with 95% CI. Only patients with one cause of pain included (n = 1,510). Scale on x-axis has been cut off at 10 for better visualisation (*note the logarithmic scale on the x-axis*). Upper limit (UL) of confidence interval for MS = 20, and for SM = 61. *DN: diabetic neuropathy, Ca: cancer, LP: lumbar pain, OP: osteoporosis; MS: multiple sclerosis, PHN: post-herpetic neuralgia, CRPS: complex regional pain syndrome; SM: Syringomyelia; CCS: carpal canal syndrome; pCVA: post-cerebrovascular accident; Alc: alcohol abuse; PSL: post-surgical lesions; PTL: post-traumatic lesions*.

Diseases that are known to be associated with NeP such as diabetes mellitus (mean LANSS score = 14.4), syringomyelia (17.5), thalamic syndrome (18.1), post-herpetic neuralgia (17.2), and CRPS type 2 (16.6) yielded higher mean LANSS scores than pathologies that are generally considered to be associated with NocP, e.g. OA (mean LANSS score = 8.7) or osteoporosis (9.2). Further support for the efficacy of the LANSS scale in detecting NeP can be found in the results of the odds ratio (OR) analysis, in which 1,580 patients reporting only one underlying cause of pain were included. Indeed, the OR of having a LANSS score ≥ 12 for patients suffering from a typical nociceptive pathology, such as OA or osteoporosis, amounted to only 0.1 and 0.2 respectively, while the OR for NeP conditions such as diabetic neuropathy, multiple sclerosis, post-herpetic neuralgia and CRPS were 3.0, 6.9, 5.4, and 3.1 respectively (Fig. 2).

However, assessment of validity can not only be determined on the basis of averages of the LANSS scores obtained for each clinical entity. In evident NeP syndromes the LANSS Pain Scale failed to correctly identify (score < 12) 25/133 of diabetic patients, 20/137 PHN patients, 6/42 patients suffering from thalamic syndrome, 9/59 patients with carpal canal syndromes, 9/75 CRPS patients, and 3/26 MS patients (all with only one pain complaint). This amounts to a total number of failures of 72/472 (15,2%). In nociceptive syndromes, the LANSS Pain Scale produced high scores (≥ 12) in 26/169 OA patients, 68/316 patients suffering from lumbar pain, and 7/26 osteoporotic patients. In these three non-neuropathic conditions, 101/511 received false high LANSS scores (19,8%). When considering the above mentioned NeP and NocP syndromes, the LANSS Pain Scale was able to correctly identify 82,4% (810/983) of patients with one pain complaint, representing 84,7% sensitivity and 80,2% specificity.

### Type of pain symptoms

The pattern in prevalence of specific subtypes of pain is clearly different between the two subgroups of patients. Data on type of pain were available for 1,245 patients belonging to the LANSS < 12 group and for 1,140 patients of the LANSS ≥ 12 group. Eighty-nine percent of the patients belonging to the LANSS ≥ 12 group reported a combination of stimulus-evoked and spontaneous pain, against only 36.2% of those from the LANSS < 12 group. In contrast, 63.2% of the patients of the LANSS < 12 group reported only spontaneous pain, against 10.2% of those in the LANSS ≥ 12 group. Isolated evoked pain complaints were rather uncommon and their prevalence was similar for both subgroups (0.6% and 0.8% of patients from the LANSS < 12 and ≥ 12 groups respectively). In addition, the various manifestations of spontaneous and provoked pain syndromes were described in different terms by the patients belonging to the different subgroups. Pain types indicative of NeP occurred much more frequently in patients with LANSS ≥ 12 than in those with a score of < 12 (Table 1). Indeed, more than twice the number of patients from the LANSS ≥ 12 group than patients from the LANSS < 12 group described their pain as a burning sensation, dysaesthesia, and electric shock sensation (Table 1). A stabbing sensation was also significantly more prevalent among the patients belonging to the LANSS ≥ 12 group compared to those belonging to the LANSS < 12 group (22.9% vs. 15.1% of patients respectively, *p *< 0.001). Most conditions of allodynia were also significantly more prevalent among patients from the LANSS ≥ 12 group than among those from the LANSS < 12 group (Table 1). Pin-prick evoked hyperalgesia was prevalent in about twice as many patients in the LANSS ≥ 12 group as in those in the LANSS < 12 group. In contrast, the occurrence of other forms of hyperalgesia was not significantly different between the two subgroups of patients (Table 1).

**Table 1:** Comparison of type of pain complaints between patients with LANSS score ≥ 12 and patients with a LANSS score < 12

	**Number (%) of patients with LANSS <12**	**Number (%) of patients with LANSS ≥ 12**	**Fischer's exact test**
**Patients with spontaneous pain complaints**	**N = 1,238**	**N = 1,131**	
Burning sensation	351 (28.4%)	788 (69.7%)	**P < 0.001**
Throbbing pain	757 (61.2%)	697 (61.6%)	P = 0.833
Paraesthesia	231 (18.7%)	178 (15.7%)	P = 0.064
Dysaesthesia	351 (28.4%)	782 (69.1%)	**P < 0.001**
Cutting, lancinating pain	378 (30.5%)	375 (33.2%)	P = 0.171
Stab sensation	187 (15.1%)	259 (22.9%)	**P < 0.001**
Electric shock sensation	155 (12.5%)	387 (34.2%)	**P < 0.001**
Other	117 (9.5%)	19 (1.7%)	**P < 0.001**
			
**Patients with complaints of provoked pain**	N = 458	N = 1,024	
Allodynia to the touch	269 (58.7%)	795 (77.6%)	**P < 0.001**
Allodynia to contact with wind	33 (7.2%)	150 (14.7%)	**P < 0.001**
Allodynia to contact with clothes	88 (19.2%)	487 (47.6%)	**P < 0.001**
Allodynia in the shower/bath	32 (7.0%)	191 (18.7%)	**P < 0.001**
Allodynia to temperature	49 (10.7%)	47 (4.6%)	**P < 0.001**
Allodynia to shaving	17 (3.7%)	62 (6.1%)	P = 0.079
Hyperalgesia in contact with needle	129 (28.2%)	650 (63.5%)	**P < 0.001**
Hyperalgesia to pressure	15 (3.3%)	22 (2.2%)	P = 0.210

### Impact of pain on sleep and daily life

Only patients belonging to the LANSS ≥ 12 group completed this part of the survey. Data on the effect of pain on sleep was recorded for 1,148 of patients belonging to this subgroup. The mean score for sleep disturbance on the VAS amounted to 5.2 (SD = 2.6) on a scale from 0 to 10, with 10 indicating a maximal sleep disturbance during the past 24 h. It is important to mention that only 8.2% of all patients completing the queries about their sleep quality failed to report any negative impact of the pain symptoms on their quality of sleep. Lumbar pain in combination with post-surgical lesions was identified as the medical condition that caused the most sleep disturbance with an average VAS score of 6.0 (SD = 2.1, n = 27). The lowest mean VAS score was found in the group of multiple sclerosis patients (mean VAS score = 4.1 SD = 2.6, n = 22). Patients with combined complaints of spontaneous and provoked pain had a mean VAS score of 5.3 (SD = 2.6; n = 1,000), whereas patients with only spontaneous complaints had a mean VAS score of 4.8 (SD = 2.4; n = 111). Hyperalgesia and allodynia caused similar degrees of sleep disturbance (mean VAS = 4.4, SD = 2.3, n = 74 and mean VAS = 4.8, SD = 2.8, n = 320 respectively). When considering the type of sleep interference, the large majority of patients reported difficulties falling asleep (60.4%), interruption of sleep (72.1%), premature awakening (60.4%) and non-restorative sleep (66.7%). In addition, 93.6% of patients reported an impact on their activities of daily living. Most of the patients reported an influence on family life (77.5%), social activities (79.8%), spare time (81.8%) and professional activities (66.1%). It should be noted that 41.4% even reported influence of their pain on all of these aspects.

### Additional investigations

On top of the clinical examination, the large majority of patients in the LANSS ≥ 12 group received complementary technical investigations to obtain final confirmation of the diagnosis of NeP. Data were available for 1,125 of the patients in the LANSS ≥ 12 group, and revealed that 1,002 of these (89.1%) were subject to one of more supplementary examinations (besides clinical examination). The most commonly performed technical investigations were electromyography (68.9% of patients), followed by radiography (59.2%), CT/MRI scan (56.2%), lab tests (51.4%), bone scan (31.4%), and a sensory evoked potential test (8.5%). Of all patients subject to additional testing, 77.8% received more than one test. Based on the results of this study, post-herpetic neuralgia is apparently perceived as the most straight-forward clinical diagnosis since only less than half of these patients (44.9%) received additional tests. In all other disorders a large majority of patients was subject to additional exams. Interestingly, our study results clearly indicate that the actual number of additional tests that were carried out is highly dependent on the underlying pathology. Most patients with carpal canal syndrome (74.2%; 23 out of 31 patients) and almost half of those suffering from diabetes (46.1%; 41 out of 89) received only one additional test, while the majority of those with another underlying pathology received two or more exams (Table 2).

**Table 2:** Percentage of patients undergoing one or more additional tests in relation to the underlying pathology in patients with LANSS ≥ 12 (n = 1,163)

	**Number of tests**						
	
**Pathology**	**0**	**1**	**2**	**3**	**4**	**5**	**6**
Diabetic neuropathy (n = 89)	6.7	46.1	19.1	10.1	11.2	6.7	0.0
Cancer (n = 27)	0.0	14.8	7.4	29.6	33.3	11.1	3.7
Lumbar problem (n = 84)	5.9	9.5	19.1	35.7	20.2	9.5	0.0
Osteoporosis (n = 3)	0.0	0.0	33.3	66.7	0.0	0.0	0.0
PHN (n = 98)	55.1	19.4	14.3	9.2	0.0	1.0	1.0
Multiple sclerosis (n = 22)	9.1	27.27	18.18	22.73	13.64	9.09	0.00
Post-traumatic lesion (n = 62)	6.4	19.3	19.3	17.7	20.9	16.1	0.0
Thalamic syndrome (n = 1)	0.0	0.0	0.0	0.0	0.0	100.0	0.0
CRPS (n = 51)	11.8	13.7	25.5	17.65	23.53	7.8	0.0
Alcohol (n = 17)	11.8	29.4	29.4	11.76	17.65	0.0	0.0
Syringomyelia (n = 6)	16.7	50.0	16.7	0.00	0.00	16.7	0.0
Post-surgical lesion (n = 34)	8.8	17.6	26.5	20.6	14.7	5.9	5.9
CCS (n = 31)	3.2	74.2	16.1	3.2	3.2	0.0	0.0
Osteoarthritis (n = 17)	11.8	5.9	23.5	17.6	23.5	11.8	5.9
Post-CVA (n = 24)	12.5	25.0	37.5	16.7	4.2	4.2	0.0
Others (n = 119)	13.4	25.2	21.8	20.2	10.9	6.7	1.7

Abbreviations: PHN: post-herpetic neuralgia; Post-CVA: post-cerebrovascular accident; CRPS: complex regional pain syndrome; CCS: carpal canal syndrome

### Pharmacological treatment of the pain

#### Previous treatment

In total, 95.1% of patients (n = 1,084) in the LANSS ≥ 12 group had received pharmacological treatment prior to enrolment in this survey. In almost all patients this (previous) treatment consisted of prescription drugs (88.5% of the patients) or a combination of prescription and over-the-counter (OTC) drugs (9.8%). Paracetamol was the most commonly prescribed drug (67.1% of the patients) followed by drugs belonging to the non-selective non-steroidal anti-inflammatory drugs (NSAID)/cyclo-oxygenase-2 specific (COX-2) inhibitors (61.4%). On the third place came the antidepressant agents (41.4%). Less common were opioids (although still taken by about 30.5% of the patients), anti-epileptic drugs (AED) in 18.0% of the patients, and finally acetylic salicylic acid (4.2%). Similar treatment patterns were found in patients consulting a GP or a specialist. However, patients treated by specialists received more treatments using AED than when treated by primary care physicians (25.5% of patients in specialist care versus 14.4% in primary care). In contrast, the use of antidepressant drugs was more common in patients seeking help of primary care physicians than specialists (44.7% vs. 34.4%).

**Figure 3:**
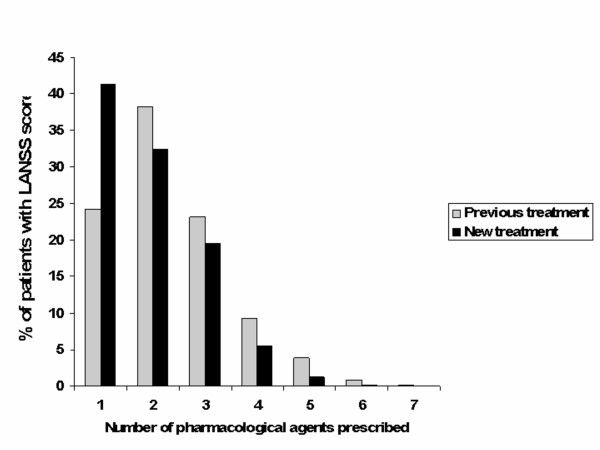
**Use of analgesic agents**. Number of pharmacological agents prescribed before and after applying the LANSS pain scale evaluation in LANSS ≥ 12 group.

Only 24.1% of the patients had previously received one drug, while 38.2% had been prescribed a combination of two drugs, and 23.1% had even received three different drugs (Fig. 3). Finally, 14.6% received a combination of four or more medications. When treated with a combination of analgesics, the most frequently used combination was paracetamol and NSAID/COX-2 inhibitors (14.6% of patients), followed by a combination consisting of NSAID/COX-2 inhibitors, paracetamol and antidepressive agents (8.9%). The use of paracetamol and NSAID was fairly similar across underlying conditions (data not shown) with about 60–75% of patients taking these drugs. In contrast, considerable variations in the prescription of opioids, AED and antidepressants were recorded across the various pathologies. Opioids were the predominant drugs in cancer pain conditions (62.8% of 47 cancer patients) as well as in patients with post-surgical lesions (44.0% of 134 patients). Antidepressant drugs were prescribed to many patients suffering from MS (56.7% of 32 patients), post-herpetic neuralgia (49.6% of 127 patients), and pain due to a cerebrovascular accident (post-CVA pain) (55.8% of 44 patients). AED were commonly taken by patients with post-CVA pain (48.8% of 44 patients), post-herpetic neuralgia (23.1% of 127 patients), post-surgical lesions (21.6% of 134 patients), and in patients suffering from diabetic neuropathy (18.6% of 160 patients).

#### Future treatment options

When questioned about which specific future treatment options they considered appropriate for their patients, physicians indicated that they considered pharmacological treatment (either starting up or continuation) in 87.6% of the patients from the LANSS ≥ 12 group (n = 1,163). Interestingly enough, physicians stated that respectively 36.6% and 17.6% of the patients would be referred for physiotherapy and for psychosocial support.

**Figure 4:**
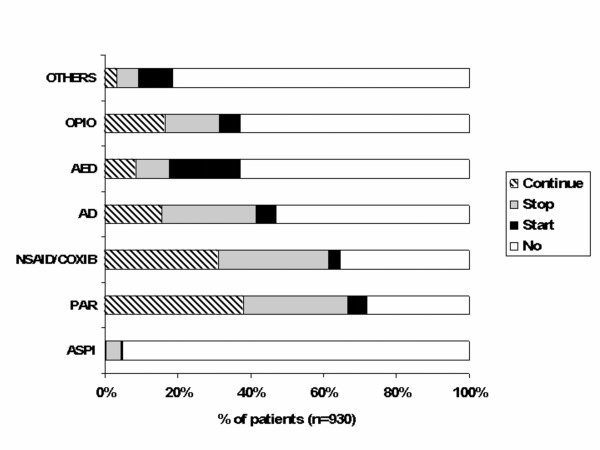
**Current and future drug treatments**. Past and future treatment : proportion of patients taking only one drug who (1) will continue this treatment, (2) stop taking it, (3) who were not taking it but will start this treatment (4) who were not taking it and will not take it as new treatment. PAR: paracetamol; AD: antidepressive agent; OPIO: opioids; ASPI: aspirin (= acetylic salicylic acid).

Compared to previous treatment regimens, physicians indicated that future treatment would consist of less drugs being prescribed concomitantly (Fig. 3): whereas only 24.1% of patients had received pharmacological monotherapy in the old regimen, 41.3% of patients would receive only one drug after physicians had filled out the LANSS questionnaire. New monotherapy would consist mainly of AED (23.0%), followed by paracetamol (4.8%). Treatment strategies before and after applying the LANSS pain scale are illustrated in Fig. 4 (n = 930). Only about half of the patients who previously received a pharmacological agent (paracetamol, NSAID/COX-2 inhibitors, opioids, antidepressive agents, or AED) would continue to receive the same drug in the future (Fig. 4). In contrast, 23.5% of patients would receive AED for the very first time (180 out of 765 AED-naive patients).

## Discussion

Progress in the further understanding of neuropathic pain (NeP) in the general population has been seriously hindered by a lack of epidemiologic research. One reason for the lack of population-based epidemiologic data has been the lack of a good case identification instrument for use in surveys. In this study the Leeds Assessment of Neuropathic Symptoms and Signs score (LANSS) was applied to investigate the management of neuropathic pain in Belgium. The LANSS scale was developed as a clinic based instrument for identifying patients whose pain is dominated by neuropathic mechanisms [11]. Although the LANSS scale has been validated in different clinical settings [11-17], it has never before been applied to such a large sample from the general population. In addition, our data collection was performed by a mixed group of general practitioners and specialists, where previous studies always relied on a limited number of investigators to administer the testing. Previous studies applied the LANSS on selected patient populations [13,18], but its usefulness was never really tested in routine clinical practice, nor in a primary care setting. However, a general population survey was recently performed with the self-complete version of the LANSS scale (S-LANSS) [19]. Finally, although this study has a local setting (Belgium), we believe that most of the results concerning diagnosis and management of NeP can be extrapolated to other neighbouring countries.

The average age of the participating patients was 58 years, and the majority were women. This is congruent with literature data showing that female gender and higher age constitute major risk factors for chronic pain [20-22].

It is generally conceived that about 25% of all chronic pain patients suffer NeP [3]. In our study however, almost half of the pain patients presented with a LANSS score ≥ 12. This unexpectedly high proportion of patients symptomatic of NeP suggests that participating physicians perhaps have preferentially included patients with NeP symptoms (recruitment bias), despite the specific request to include the first 15 pain patients presenting, irrespective of their specific complaints. However, the objective of our study was not to estimate the prevalence of NeP, but rather assessing the diagnosis and management of NeP in Belgian practice. For purely practical reasons, it was decided not to complete the additional questionnaires in patients with LANSS scores < 12. This is without any doubt a methodological limitation of the study, since it could have been interesting to compare the treatment and functional status of both neuropathic (NeP) and nociceptive (NocP) pain patients in Belgium. However, demanding that additional questionnaires would be completed for *all *participating patients would have significantly increased the physicians' work load, perhaps further diminishing their preparedness to include sufficient number of patients.

### The use of a screening scale as instrument for helping to diagnose NeP

Bennett [11] reported a sensitivity and specificity of 83% and 87% respectively for the LANSS scale. The purpose of this study was not to validate the LANSS scale, but rather to provide physicians with an instrument for assisting them in the diagnosis of NeP. Average LANSS scores in conditions associated with NeP such as diabetes and multiple sclerosis were well above 12, while non-neuropathic conditions such as OA scored mean values below 12. Pain symptoms known to be associated with NeP such as allodynia, hyperalgesia, shooting pain, electric shock sensations, and burning and throbbing pain were significantly more prevalent in patients with a LANSS pain score ≥ 12. The results of the odds ratio analysis provide an even stronger support for the relationship between a LANSS score of ≥ 12 and NeP conditions. Limited number of patients suffering from typical neuropathic pathologies ended up with low LANSS-scores (15,2%), while patients suffering from probable nociceptive pain conditions displayed high LANSS-scores in 20,3% of cases. These figures provide important additional information regarding the sensitivity and specificity of the LANSS-scale in patients displaying one type of pain within our particular setting. With the suggested cut-off score of 12, the LANSS Pain Scale displayed a good sensitivity (84,7%) and only a slightly lower specificity (80,2%). Sensitivity and specificity is therefore largely comparable to those previously reported in other studies [11,13,14,16,18]. This is a strong indication that the LANSS-scale can be used in larger population samples and by different groups of physicians. Nevertheless, not all patients with a typical neuropathic condition actually do suffer from neuropathic pain symptoms. It is estimated that 75% of syringomyelia patients and 20–24% of diabetes patients have NeP [4,23]. Taken together, this data suggests that using a screening scale such as the LANSS may prove a useful tool for discriminating between nociceptive and neuropathic pain in routine clinical practice. The routine application of the LANSS scale could assist in modifying the analgesic approach to these patients.

It should be noted that distribution of patient populations and pain characteristics did not differ significantly between GP's and specialists. This should perhaps be considered a somewhat typical Belgian finding, since general practitioners have no major gate-keeping role in the referral of patients to specialist care. In other countries (e.g. The Netherlands) GP's tend to be much more in control of this referral process, which probably leads to significantly different patient populations and pain characteristics in specialists' reception compared to those in GPs'. In addition, no significant differences were observed in the obtained LANSS-scores, which proves that this scale can be used by a diverse group of physicians.

Post-herpetic neuralgia and diabetic neuropathy were the most common underlying diagnoses in patients with LANSS score ≥ 12 for whom one cause of pain was diagnosed. This is in line with data from other investigators [3,5,20], and further supports the usefulness of the LANSS scale for detecting NeP. The observation that the non-neuropathic condition OA has been diagnosed in an appreciable proportion of (putative) NeP patients is consistent with previously published data that NeP patients are disproportionably affected by OA [20]. In addition, considering the relatively high average age of the patients (58 years), it can be expected that many of these may have OA concomitantly with another (non-OA related) NeP condition. This is clearly reflected by the fact that almost 40% of the patients with a LANSS score ≥ 12 present with multiple underlying causes of pain, some of which may be of non-neuropathic origin. Indeed, physicians were requested to record any possible cause of underlying pain for all patients, regardless of whether or not it might be related to NeP.

Considering the observation that almost 90% of the patients with a LANSS score suggestive for the possible presence of a neuropathic pain syndrome, still received additional technical investigations, a more widespread use of these types of screening scales may perhaps decrease the need of additional medical exams and speed up the implementation of neuropathic-specific treatment regimens (although a definite or probable diagnosis of neuropathic pain surely implies a proof of damage to the somatosensory system by neuroimaging or electrophysiology). It is of importance that NeP should be treated promptly, because the longer the delay in appropriate therapy, the lower the proportion of patients in whom the NeP is relieved [3]. Fewer additional investigations would significantly decrease the financial burden upon patient and healthcare system. Patients with painful neuropathies incur health care costs that are three times higher than those without NeP [20].

### Impact of NeP on sleep and daily life

The negative impact of chronic pain including NeP on Quality of Life (QoL) is well documented. Chronic pain adversely affects overall health, daily activities and productivity at work, and is an important predisposing factor for depression [21,24-26]. Daily chronic pain relates stronger to poor health than chronic disease or age [25]. Our study results are consistent in that the majority of (putative) NeP patients reported their pain affecting social, family and professional life, and spare time. Likewise, the negative impact of the pain on sleep reported in our survey is congruent with data showing that the large majority of chronic pain patients report a considerable degree of sleep disturbance [27]. However, the association between sleep and chronic pain may be complex [27-29]. Firstly, chronic pain results often in mood disturbances, anxiety and depression [6], disorders that in turn negatively impact sleep [30]. Secondly, lack of sleep decreases pain thresholds and leads to increased pain perception [31,32], and/or diminishes the patient's ability to cope with pain. Furthermore, sleep deprivation is associated with a decreased QoL along with a poor mental and somatic health [33,34]. Hence, the sleep disruption caused by chronic pain may predispose patients for additional morbidity. Consequently, alleviating NeP will likely result in improved QoL. A concomitant increase in QoL scores including sleep, activity and mood is observed following a significant relief of the NeP [4,35].

### Treatment of NeP in Belgian routine practice

Recent recommendations for the treatment of NeP suggest the use of AED (gabapentin, carbamazepine and others), tramadol, the 5% lidocaine patch or antidepressants as a first-line therapy for NeP [8-10]. It should be borne in mind however, that no antineuropathic drug is effective against all manifestations of NeP [4], and that patients with identical NeP conditions may respond quite differently to the same pharmacological approach [36]. Furthermore, potential side effects associated with antineuropathic drugs should be taken into account when deciding upon the appropriate treatment. Patients who are refractory to any first-line therapy may benefit from other AED or antidepressant drugs, the combination of several anti-neuropathic drugs, or may have to be referred to a multidisciplinary pain centre [8,10].

The data obtained in our study shows that in Belgium NeP is still mainly managed with conventional analgesics such as paracetamol and NSAIDs, instead of the more effective anti-neuropathic drugs. A widespread use of conventional analgesics in the treatment of NeP has been reported by others [4,5,20]. Gilron et al. [5] reported that 25% of neuropathic pain patients in their survey had never tried any antineuropathic drugs, despite 73% of respondents complaining of inadequate pain control; almost half of NeP patients (47%) took paracetamol or NSAID. It has been suggested that healthcare providers might feel more comfortable in prescribing traditional analgesics for the treatment of pain, irrespective of its origin [20]. The limited efficacy of these conventional analgesics in the treatment of NeP may be reflected in the high prevalence of (conventional drug type) combinations reported in our study: three quarters of the patients with a LANSS score ≥ 12 who received pharmacological treatment, initially received at least two different analgesic drugs, while over one third had even received combinations of three or more drugs. However, upon completing the LANSS questionnaire, a treatment simplification with a reduction in the number of drug combinations was apparent. Especially AED had become the drugs of choice as monotherapy. This may reflect some increased awareness among physicians about NeP. It is tempting to speculate that the request to employ the LANSS scale as an aid in the diagnosis of chronic pain may have brought the topic of NeP and its specific treatment options to the attention of the physicians.

## Conclusion

In conclusion, our study results confirm previous reports that NeP is under-recognised and often not treated adequately in daily practice, and that it has a considerably negative impact on QoL. NeP patients are treated mainly with combinations of conventional analgesics rather than the more effective antineuropathic drugs, indicating that physicians often do not follow the guidelines for NeP. Clearly, awareness among physicians about NeP and the availability of diagnostic tools should be augmented, and the importance of applying appropriate treatment regimens tailored to the individual patient's needs should be more strongly emphasised. The results of this study indicate that the LANSS-scale is a useful tool to discriminate patients with a neuropathic pain component and to assist in modifying the analgesic approach to these critical pain syndromes.

## Competing interests

The author(s) declare that they have no competing interests.

## Authors' contributions

GH participated in the design of the study, served as a principal investigator and drafted the manuscript. EM participated in the design of the study and served as principal investigator in this study. PDC conceived the study, coordinated the practical aspects of this study project and helped to draft the manuscript. All authors read and approved the final manuscript.

## Pre-publication history

The pre-publication history for this paper can be accessed here:


